# Optimisation of the two-dimensional gel electrophoresis protocol using the Taguchi approach

**DOI:** 10.1186/1477-5956-2-6

**Published:** 2004-09-09

**Authors:** Guennadi A Khoudoli, Iain M Porter, J Julian Blow, Jason R Swedlow

**Affiliations:** 1 Division of Gene Regulation and Expression, School of Life Sciences, Wellcome Trust Biocentre, University of Dundee, Dow Street, Dundee DD1 5EH, UK

**Keywords:** *Xenopus *egg extract, chromatin, cell cycle, two-dimensional gel electrophoresis, proteomics, Taguchi method, optimisation

## Abstract

**Background:**

Quantitative proteomic analyses have traditionally used two-dimensional gel electrophoresis (2DE) for separation and characterisation of complex protein mixtures. Among the difficulties associated with this approach is the solubilisation of protein mixtures for isoelectric focusing (IEF). To find the optimal formulation of the multi-component IEF rehydration buffer (RB) we applied the Taguchi method, a widely used approach for the robust optimisation of complex industrial processes, to determine optimal concentrations for the detergents, carrier ampholytes and reducing agents in RB for 2DE using commercially supplied immobilised pH gradient (IPG) gel strips.

**Results:**

Our optimisation resulted in increased protein solubility, improved resolution and reproducibility of 2D gels, using a wide variety of samples. With the updated protocol we routinely detected approximately 4-fold more polypeptides on samples containing complex protein mixtures resolved on small format 2D gels. In addition the pI and size ranges over which proteins could be resolved was substantially improved. Moreover, with improved sample loading and resolution, analysis of individual spots by immunoblotting and mass spectrometry revealed previously uncharacterised posttranscriptional modifications in a variety of chromatin proteins.

**Conclusions:**

While the optimised RB (oRB) is specific to the gels and analysis approach we use, our use of the Taguchi method should be generally applicable to a broad range of electrophoresis and analysis systems.

## Background

During cell cycle progression different functional protein complexes associate with and dissociate from chromosomal DNA [1,2]. We have taken a proteomic strategy to identify and then characterize proteins that are bound to chromatin at defined stages of the cell cycle in cell free extracts derived from *Xenopus *eggs. The combination of 2D gel electrophoresis (2DE) and Mass Spectroscopy (MS) are powerful tools for this analysis.

2DE is capable of resolving thousands of proteins in a single separation procedure [3]. Development of immobilised pH gradients (IPG) coupled with pre-cast gradient polyacrylamide gels and introduction of new sensitive fluorescent stains have considerably simplified and greatly improved the capacity, sensitivity and reproducibility of 2D gels. These recent technological advances do not however eliminate a number of difficulties associated with the separation of proteins by 2DE. One major problem is the solubilisation of protein mixtures during isoelectric focusing (IEF), (reviewed in [4]). In addition, reduction and alkylation of protein samples for 2DE has not yet been fully optimised [5,6]. As a consequence, conventional approaches for protein solubilisation and modification do not reliably provide the best samples for electrophoresis.

Good solubilisation of protein samples is critical for high performance 2D electrophoresis and there is a wide range of protein solubilisation cocktails reported in the literature. However we have not found any systematic studies reporting optimal concentrations of critical ingredients, possibly because conventional approaches to optimisations are very time consuming: varying all the possible components in turn and in combination is quite laborious. There are, however, methods for reducing the complexity of multi-parametric matrices. The Taguchi method, has been widely used for several decades in the development of industrial processes and recently found its way to the area of life sciences [7-10]. The conventional optimisation experiments require independent testing of each variable in turn. For example, testing the effect and interaction of four different reaction components, each at three separate concentration levels, would require experiment with 81 (i.e. 3^4^) separate reactions. Using Taguchi approach the same task can be accomplished in the experiment with just nine reactions.

To find the optimal and most robust conditions for 2DE, we applied a modified Taguchi method [9] for the formulation of the rehydration buffer (RB) used to solubilise and run protein mixtures during IEF. We also optimised the sample reduction and alkylation procedure traditionally performed after IEF step. The resulting protocol, substantially improved the solubility and resolution of protein mixtures derived from a variety of sources on 2DE.

## Results

### Choosing components for optimisation of RB

Rehydration buffers for IEF generally consist of chaotropes (urea, thiourea), detergent(s), reducing agent(s) and carrier ampholytes (reviewed in [11]). The standard formulation of RB (sRB) contains 8 M urea [12]. However the combination of chaotropes, 7 M urea and 2 M thiourea, was reported to produce better 2D images with an immobilised pH gradient (IPG) compared to 8 M urea alone [13] and this mix was chosen as the basis for all subsequent rehydration solutions.

**Figure 1:**
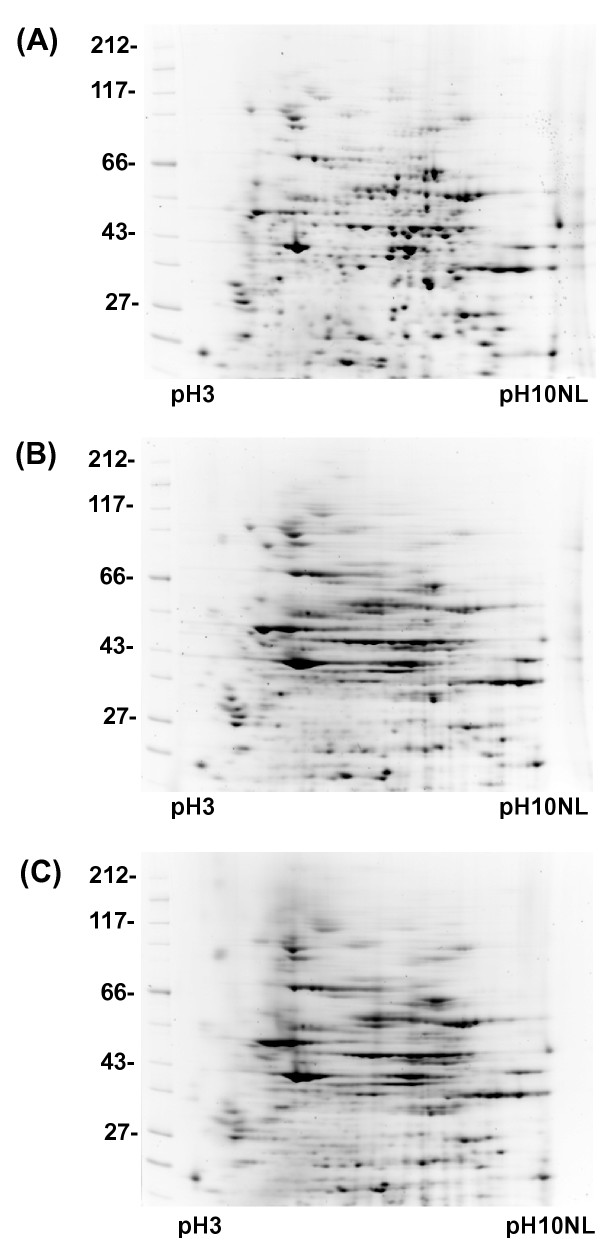
The effect of different reducing agents on protein resolution in 2D gels. 50 μg aliquots of total *Xenopus *egg extract were dissolved in RB containing 7 M urea, 2 M thiourea, 4% CHAPS, 0.5% ampholytes and different reducing agents. 2DE separation was conducted as described in Materials and Methods. (A) 20 mM DTT, (B) 2 mM TBP, (C) 10 mM TCEP.

During IEF, proteins must be maintained in a fully reduced state. Three reducing agents: DTT, TBP and TCEP were tested in identical conditions with the same protein sample using an RB containing 7 M urea, 2 M thiourea, 4% CHAPS, 0.5% ampholytes and either 20 mM DTT, 2 mM TBP or 10 mM TCEP. 50 μg pellets of *Xenopus *egg proteins were solubilised in each RB and separated by 2DE (Figure 1). Both TBP and TCEP reduced focusing in our gel system. The best focusing was achieved in the RB containing DTT, so this compound was selected for all successive optimization experiments.

Detergents in RBs help to prevent protein interaction and aggregation, and their properties are critical for protein solubilisation. The increasing number of proteins detected in 2D gels was reported when different zwitterionic detergents were added to solubilisation cocktails [14-16]. We therefore decided to optimise the combination of two widely used detergents, CHAPS and ASB14 [17,18].

The addition of carrier ampholytes enhances solubility of individual proteins as they approach their isoelectric points. They also produce an approximately uniform conductivity across a pH gradient without affecting its shape. For these reasons, we also optimised the concentration of carrier ampholytes within the solubilisation buffer.

The concentration ranges of components used in one representative optimisation experiment are presented in Table 1. To accommodate increased carrier ampholyte concentrations, we increased the length of focusing time during electrophoresis. We found that a one step fast ramping gradient between 250–5500 V after the initial phase of 30 min at 250 V worked well for 7 cm IPG strips of different pH ranges (pH 3–10, pH 4–7, pH 6–11). The duration of the IEF step was dependent on both sample conductivity and protein loading, producing good results if performed for a total of more than 33000 volts-hours. The actual voltage was limited since the current was restricted to 50 μA/strip. This total value of volt-hours was enough to complete focusing in samples with the highest carrier ampholyte concentrations.

**Table 1:** Set up of the Taguchi optimisation experiment presented in Figure 2 with total number of spots detected in each gel.

Buffer*	Ampholytes(%)	CHAPS(%)	ASB14(%)	DTT(mM)	Spot number**
1	0.5	0.5	0.4	20	**361**
2	0.5	1.0	0.8	40	**339**
3	0.5	2.0	1.6	80	**339**
4	1.0	0.5	0.8	80	**296**
5	1.0	1.0	1.6	20	**351**
6	1.0	2.0	0.4	40	**355**
7	2.0	0.5	1.6	40	**319**
8	2.0	1.0	0.4	80	**327**
9	2.0	2.0	0.8	20	**299**

* – all buffers contained 7 M urea, 2 M thiourea, 10 mM Tris

** – number of spots detected, using Phoretix 2D Pro imaging software

After IEF, the focused gel is prepared for SDS-PAGE, usually by incubating consecutively in two equilibration buffers containing DTT or iodoacetamide (IAA) respectively. IAA serves to alkylate reduced cysteine residues and prevent their modification during and after SDS-PAGE. There is evidence that this protocol is not very efficient as the SDS in equilibration solution interferes with IAA alkylation. Variable alkylation can cause a substantial number of artefactual spots on 2D gels [6,19]. One of the ways to overcome the problem is to alkylate protein mixtures in solubilisation buffer before the IEF. However, the presence of thiourea in the RB prevents effective protein alkylation by IAA [5]. Acrylamide is an alternative alkylating agent, and is used widely for protein modification in MS studies. To alkylate cysteines we adjusted acrylamide concentration in RB to 60 mM after solubilisation of protein pellets for 2 hours in the presence of the indicated amount of DTT. The acrylamide treatment was also repeated after IEF as part of the equilibration procedure (see Materials and Methods).

### Taguchi optimisation of RB

**Figure 2:**
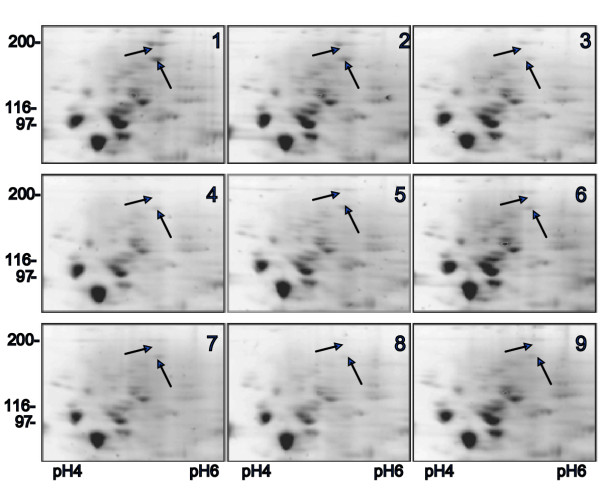
2D gel images from a representative optimisation experiment. Nine rehydration buffers were tested according to Table 1. Each buffer was used to solubilise 50 μg whole Xenopus egg extract and each sample was run on identical gels under identical conditions. (1–9) are images of high molecular weight regions of 9 gels with arrows pointing to some spots whose intensity and focusing pattern changed considerably between different RB compositions. Total numbers of detected spots in individual gels are presented in Table 1.

Aliquots of total *Xenopus *egg extract containing 50 μg of protein were dissolved in rehydration buffers formulated according to the L9 orthogonal Taguchi array shown in Table 1 and then separated by 2DE. The resulting 2D gels exhibited different degrees of focusing and spot presentation especially in the high molecular weight region of the gels (Figure 2). The number of spots detected by commercial image analysing software in each individual gel was used to calculate the Taguchi's *SNR *values for each level of a given component. The signal-to-noise ratio (*SNR*) function is a statistical measure of performance and takes into account both the mean and variability. In its simplest form, the *SNR *is the ratio of the mean (signal) to the standard deviation (noise). While there are many different possible *SNR*s their simple interpretation is always the same: the larger the *SNR *the better. We used the Taguchi *SNR *function that is most applicable to the situation where the highest yield of optimised process is desirable (see Materials and Methods).

**Figure 3:**
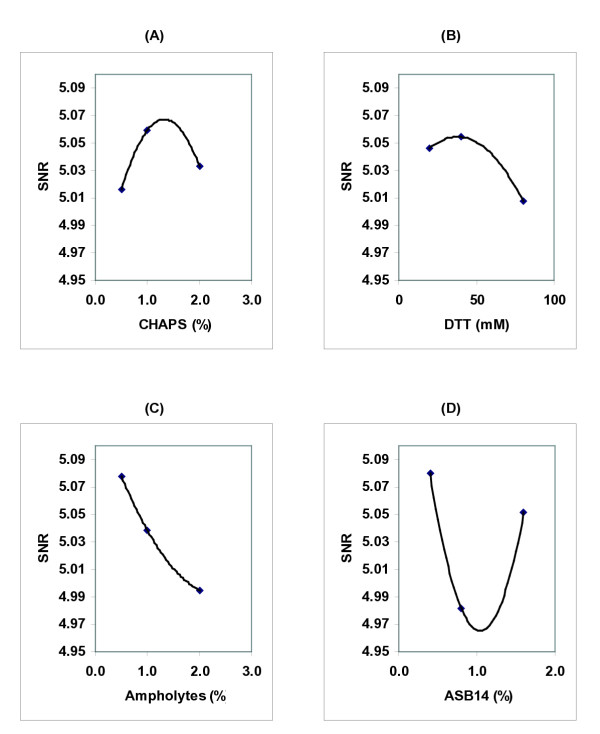
Effects of reaction components on *SNR *functions in a representative experiment. The Taguchi calculations were carried out using total number of spots detected by imaging software in 2D gels (Table 1). The optimal concentration of each component corresponded to the highest value of the *SNR *function (a–d).

Figure 3 demonstrates the *SNR *graphs from a representative optimization experiment. For two variables in this experiment, CHAPS and DTT, the *SNR *functions had a maximum within the analysed range and highest *SNR*s were achieved at 1.32% CHAPS and at 34 mM DTT (Figure 3A,3B). By contrast the highest *SNRs *were achieved with the lowest concentration of ampholytes and ASB14 (Figure 3C,3D).

To analyse reproducibility of our approach we repeated experiments three times and determined optimal concentrations of RB components as (1.20 ± 0.18)% CHAPS, (43 ± 12) mM DTT, 0.25% ampholytes and 0.4% ASB14 (the last two are the lowest concentrations used in our optimisation experiments).

The *SNR *response for ASB14 suggests there may be two concentrations that increase the number of detected spots. While we chose detected spot number as a general reporter of RB performance, other factors such as spot circularity, streaking, spot intensity, etc are also critical. We noted that concentrations of ASB14 slightly higher than 2.0% induced significant streaking and spot shape changes leading to a reduced number of detectable spots (see Additional file 1). This suggested that concentrations of ASBB14 near 2.0% could not perform robustly, i.e. small changes would have deleterious effect on 2DE.

Figure 3C and 3D suggest that low concentrations of ampholytes and ASB14 may improve detected spot number. To determine if we achieved optimal concentrations for these components, we assayed whether further reducing the amount of these components might increase detected spot number and further improve 2DE performance. Using concentrations of 0.1% – 0.4% ASB14 and 0.05% – 0.25% ampholytes we found no significant changes in detected spot number (see Additional file 1). Thus, the chosen concentrations of ASB14 and ampholytes gave a robust performance and we therefore considered them to be optimised for our 2DE system.

**Figure 4:**
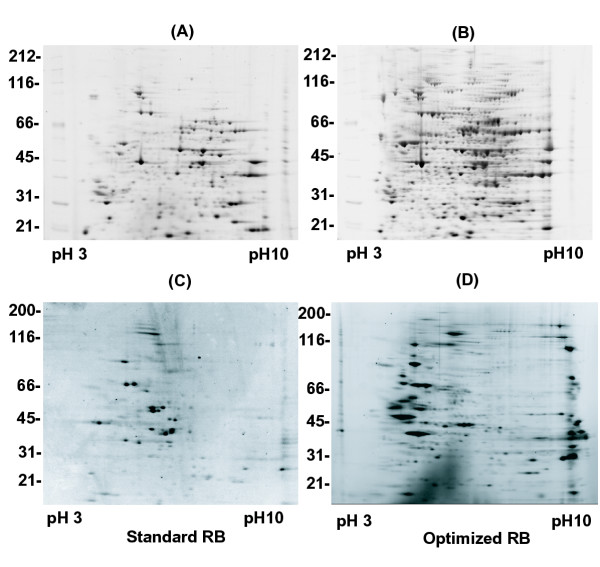
2DE of two different sets of proteins in sRB (A, C) and oRB (B, D). 50 μg of total *Xenopus *egg extract (A, B) or 25 μg of protein eluted from mitotic chromosomes assembled for 30 min in mitotic *Xenopus *egg extract (C, D) were dissolved in sRB (8 M urea, 4% CHAPS, 0.5% ampholytes, 20 mM DTT) and oRB (7 M urea, 2 M thiourea, 1.2% CHAPS, 0.4% ASB14, 0.25% ampholytes, 43 mM DTT, 30 mM Tris) and separated as described in Materials and Methods. Spots detected: (A) 425, (B) 659, (C) 112, (D) 350.

To confirm that the correct choice was made for the optimal ASB14 concentration we compared 2D gels of total *Xenopus *eggs proteins separated with sRB (8 M urea, 4% CHAPS, 0.5% ampholytes, 20 mM DTT) and with oRB (7 M urea, 2 M thiourea, 1.2% CHAPS, 0.4% ASB14, 0.25% ampholytes) (Figure 4A,4B). We observed at least a 50% increase in detected spot number in oRB (659 spots) compared to the standard buffer composition (425 spots).

To extend our analysis we have evaluated the performance of oRB with a variety of different samples. Chromatin associated proteins are often enriched in lysine and arginine residues that mediate interactions with DNA. When we analysed a preparation of mitotic chromosome proteins [20] using sRB, we noted a distinct lack of basic and high molecular weight polypeptides detected on the gel (Figure 4C). 2DE of chromosome associated proteins with oRB revealed a large number of basic proteins, as well as many high molecular weight polypeptides, including known high molecular weight chromosome components like DNA topoisomerase II and condensin [21] (Figure 4D). Similar performance was noted with preparations of human nucleolar proteins, mouse and shrimp mitochondria (data will be presented elsewhere). We therefore concluded that our optimised 2DE methodology could be successfully applied to a wide variety of samples.

### Analysis of post-translational modifications by 2DE

**Figure 5:**
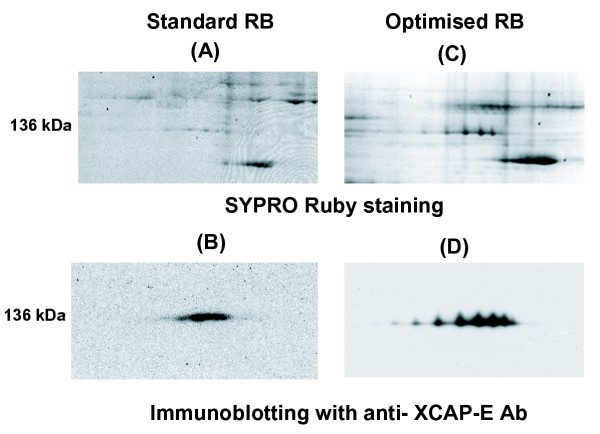
Effect of different rehydration buffers on quality of western blotting analysis of 2D gels. 25 μg of mitotic chromosomes eluates were separated on pH6-11 IPG strips using sRB (A, B) or oRB (C, D) as in Figure 4. 2D gels were stained with SYPRO Ruby (A, C) or immunoblotted using anti-XCAP-E antibody (B, D). The protein spots seen on the top right of (A) were not reproducible and were perhaps due to poor alkylation. The spot pattern observed in (C) was reproducible.

As a test of the use of an oRB, we characterised a series of well-known chromatin proteins whose function is regulated during the cell cycle. Our improved RB and revised alkylation procedure eliminated ambiguities in immunoblots of high molecular weigh proteins and revealed specific modifications that have not been described previously. XCAP-E (M.W. 140 kDa), a component of condensin, a protein complex involved in the assembly of mitotic chromosomes [21], was analysed in mitotic chromatin eluates. With sRB, 2D gels stained with SYPRO-Ruby or immunoblotted with an anti-XCAP-E antibody produced only a smear, suggestive of poor solubilisation or focusing (Figure 5A,5B). The same sample was resolved into 7 distinct spots by oRB and acrylamide alkylation (Figure 5C,5D).

Phosphorylation is one of the post-transcriptional modifications that can change the pI of proteins. Previous analysis has not detected significant phosphorylation in XCAP-E and treatment of these samples with phosphatase produced no change in the 2DE pattern (data not shown). We are currently exploring the nature of this apparently specific modification. Regardless, an optimised 2DE methodology can reveal multiple forms of proteins, even relatively large ones that are traditionally poorly resolved.

**Figure 6:**
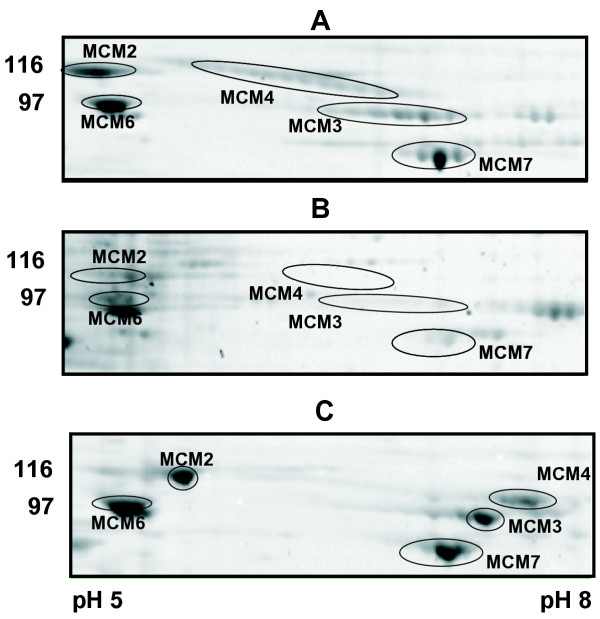
Cell cycle dependent association of replication licensing complex with chromatin. Proteins eluted from chromatin assembled for 30 min in *Xenopus *egg extracts were separated on pH 3–10 2D gels and stained with SYPRO Ruby. In control assembly reaction MCM2,3,4,6 and 7, were detectable in eluates as multiple spot chains (A). In the presence of geminin, association of MCMs with chromatin was prevented (B). The treatment of control chromatin eluates with λ-phosphatase changes the MCMs presentation in the 2D gel (C).

The 6 mini chromosome maintenance proteins MCM2-7, which are a central component of the replication licensing system, are bound to *Xenopus *chromatin from the end of mitosis and are released during S-phase [22,23]. The six proteins (M.W. ~90–105 kDa) were identified by 2DE from replicating chromatin eluates using immunoblotting analysis and MALDI-TOF (data not shown). Figure 6 shows 2DE images of MCM2, 3,4,6 and 7 eluted from replicating *Xenopus *chromatin at the beginning of S-phase in a control experiment (Figure 6A) and in extract treated with geminin, which prevents loading of MCM2-7 onto chromatin (Figure 6B) [24-26]. The treatment of chromatin eluates with λ-phosphatase changed the distribution of spots corresponding to MCM2, 3 and 4 whereas MCM6 and MCM7 spots remained mostly unchanged (Figure 6C). With the improved resolution of 2DE provided by oRB and sample treatment, the analysis of post-translational modifications of relatively large proteins is now possible.

## Discussion

In this study we successfully applied a modified Taguchi strategy [9] to optimise conditions for 2DE. An optimal formulation of the rehydration buffer used to dissolve proteins and run IPG strips in the first dimension was determined using the Taguchi experimental design based on an orthogonal L9 array (Table 1).

We observed that the resolution of proteins over 100 kDa depended heavily on the composition of the RB and were able to define a recipe that provides significantly improved 2DE performance for many different samples.

Our data demonstrated that the combination of different detergents in the rehydration solution improved the solubility and resolution of proteins on 2D gels, though their optimal concentrations in the mix may be very different from the ones published for mono-detergent solutions. In addition, we found that the concentration of ampholytes has the biggest effect on *SNR *variation and that the minimal values of 0.25% produced the highest *SNR*. However, different regions of the 2D gel responded differently, with proteins <30 kDa becoming less well resolved at low ampholyte concentrations. While further reduction of ampholyte concentration in RB may be beneficial for the resolution of high molecular weight proteins, this should be performed only when resolution of low molecular weight proteins is not necessary. Our results therefore suggest a method of significantly improving 2DE performance, although we cannot specify a single, universally applicable electrophoresis protocol.

To verify the applicability of our oRB for different protein samples we applied standard and optimal conditions for the separation of protein fractions which were eluted from chromosomes reconstituted in *Xenopus *egg extracts [27,28], whole *Xenopus *egg extract, human nucleoli and mouse and shrimp mitochondria. The amount of protein generated in these experiments is limited and complete solubilisation of the samples is an important issue for 2DE and MS analysis. Use of our oRB has improved the resolution and detection of 2DE and increased the amount of protein we can provide for MS. To date, we have not observed any dependence of the composition of RB on the source of protein. Within our lab, where we use one type of gel and one gel analysis system, we now have a standard composition for RB that appears to work with all samples.

Our optimised composition of RB is determined by scoring the number of spots observed employing specific commercial IPG gel strips, pre-cast SDS-PAGE gels, and commercially available 2DE analysis software. It is likely that the optimal composition of RB depends on each of these parameters. For instance, a different 2DE spot finding algorithm might find a slightly different set of spots, especially in the extreme regions of the gel, and thus change the optimal composition of RB determined by the Taguchi method. Moreover, spot number does not reflect other spot characteristics, such as intensity, circularity, etc. The use of a single parameter for the performance evaluation is a basic tenet of the Taguchi approach and the choice of it defines the result of an optimisation experiment. We therefore used a single performance criterion as the prime determinant for oRB, and incorporated other characteristics to refine our choice of oRB constituents.

Therefore our most important findings are: (1) combinations of zwitterionic detergents, appropriately optimised, can provide improved solubilisation of proteins for 2DE; (2) the concentration of carrier ampholytes must be optimised; (3) the use of a technique like the Taguchi method can rapidly and relatively easily determine an optimal combination of RB components for 2DE. Whereas systematic evaluation of all possible combinations in a multi-component system is prohibitively costing and time consuming, the Taguchi method provides a systematic assessment of a systems behaviour that is reasonably straightforward to perform.

Our results also extend the power of 2DE in the analysis of post-translational modifications. For subsequent MS identification of proteins and characterisation of post-translational modifications, difficulties with differential solubility and focussing must be resolved. The oRB addressed these issues and substantially improved the resolution of protein isoforms, including those larger than 100 kDa.

## Conclusions

We have successfully applied the Taguchi method to optimise the complex composition of a rehydration buffer used in 2DE. The strategy greatly reduced the number of experiments required compared to classical designs, reduced cost, and has produced a substantially improved 2DE RB.

## Materials and methods

### Chemicals and equipment

Immobiline DryStrip 7 cm gels and carrier ampholytes of different pH ranges were purchased from Amersham Pharmacia Biotech UK Ltd. The Protean IEF cell from BioRad (Bio-Rad Laboratory Ltd., Hemel Hempsted, UK) was used to run the isoelectric focusing separation. For the second dimension, the focused IPG strips were loaded on precast Novex ZOOM gels (Invitrogen, Ltd, Paisley, UK) and run with NuPage MOPS SDS buffer as instructed by the manufacturer.

The zwitterionic detergent ASB14 was obtained from Calbiochem (Merck Biosciences, Ltd., Nottingham UK). Urea of AristaR grade and other general chemicals of AnalaR grade were purchased from BDH (Merck House, Poole, Dorset, UK). SYPRO Ruby (Molecular Probes, Leiden, The Netherlands) was used to stain 1D and 2D gels according to manufacture recommendations. The images of stained gels were acquired by a Fujiimager LAS1000 using the Dark Reader trans-illuminator (Clare Chemical Research, Inc., Dolores, USA). Spot detection, matching, quantification and analysis were carried out using Phoretix 2D Pro software (Nonlinear Dynamics Ltd, Newcastle, UK). The antibody against XCAP-E (R5-5) was kindly supplied by T. Hirano [21,29].

### Sample preparations

*Xenopus *egg extract preparation, chromatin reconstitution, and elution of chromosomal proteins was carried out as previously described [27,28,20]. For IEF, methanol/chloroform precipitated samples containing 50 μg protein (or as otherwise stated) were solubilised either in the standard rehydration buffer (sRB: 8 M urea/4% CHAPS/0.5% ampholytes/20 mM DTT) or RBs prepared according to the Taguchi orthogonal array (Table 1). The composition of optimised rehydration solution was as follows: 7 M urea/2 M thiourea/1.2% CHAPS/0.4%ASB14/0.25% ampholytes/43 mM DTT/30 mM Tris base). Solubilisation was carried out on a vibra-shaker for 2 hours at room temperature. To alkylate proteins before IEF, freshly prepared 9 M acrylamide in water was added to each sample to a final concentration of 60 mM and incubation continued for another 1.5 hours at room temperature. At the end of the incubation, samples were spun for 10 min at 16000 × *g *in a micro-centrifuge and transferred to rehydration chambers. The dry IPG strips were allowed to re-swell in RBs over night before IEF separation. For samples that were prepared in sRB solubilisation was continued for 2 hours without alkylation before starting the IPG re-swelling.

### Electrophoresis and spot detection

Isoelectric focusing was performed using a two phase protocol: (1) 250 V for 30 min and (2) 250 – 5500 V fast ramping voltage gradient to accumulate 33000 total volt-hours. The focused IPG strips were subjected to additional reduction and alkylation treatment before the second dimension SDS-PAGE. The strips were equilibrated for 20 min in 25 mM DTT dissolved in 6 M Urea/2% SDS/30% glycerol/50 mM Tris HCl pH8.8 and then alkylated by incubating with 360 mM acrylamide for 20 min in the same buffer. Equilibrated IPG strips were applied to precast Novex 4–12% Zoom gels and run at room temperature for 1 hour at 200 V. SYPRO Ruby stained gels were imaged and the total number of spots in individual gels was determined by Phoretix 2D Pro imaging software. The resulting data were visually inspected to remove background artefacts. The number of spots identified in each individual gel was used in the Taguchi calculations.

### Taguchi experimental design

In the Taguchi method, variables under optimisation are arranged into orthogonal array (Table 1, L9 orthogonal array for the representative experiment). With 2DE, each column would correspond to individual buffer components, and each row would represent individual IEF rehydration buffer. Each component is taken at three defined concentration (A, B and C), covering the range where its effect can be determined. The number of spots detectable in 2D gels prepared with individual RB compositions (the yield of trials) is used to evaluate the effect of the components. This is done by calculating Taguchi's signal to noise ratios (*SNR*) for each component. The goal with 2DE is to maximise the numbers of detectable spots. For this aim G. Taguchi designed the following *SNR *function:



where *SNR *is signal to noise ratio, *n *is a number of trials with given concentration and *Y*_*i *_is the yield in correspondent trials [9,30]. To calculate the *SNR *for 1% CHAPS, for example, we used total spot numbers in gels 2, 5 and 8 (*n *= 3), where CHAPS was present at 1% level (Table 1). A second order polynomial fit was employed to calculate the concentration corresponding to the maximum of *SNR *if it was present on a graph.

### Mass spectrometry

The spots of interest were excised from 2D gels and were subjected to MALDI-TOF or LC/MS-MS analysis in the Post-Genomics and Molecular interactions Centre of University of Dundee.

## List of abbreviations

RB – rehydration buffer, sRB – standard rehydration buffer, oRB – optimised rehydration buffer, *SNR *– signal to noise ratio, TBP – tributylphosphine, TCEP – tris(2-carboxyethyl)phosphine hydrochloride, DTT – dithiothreitol, IAA – iodoacetamide, IPG – immobilised pH gradient, MCM – mini-chromosome maintenance proteins, PTM – post-translational modifications.

## Competing interests

None declared.

## Authors' contributions

GAK designed and carried out optimisation experiments, 2DE analysis of *Xenopus *licensing complex and human nucleolar proteins and drafted the manuscript. IMP carried out 2DE analysis of *Xenopus *condensin complex and mitochondrial extracts from mouse and shrimp cells. JJB participated in the design of the study and coordination. JRS conceived of the study, and participated in its design and coordination. All authors read and approved the final manuscript.

## Supplementary Material

Additional File 1 2DE response to variations of ASB14 and ampholytes concentrations. 50 μg aliquots of total *Xenopus *egg extract were dissolved in RBs containing 7 M urea, 2 M thiourea, 1.2% CHAPS, 43 DTT and variable amount of ASB14 and ampholytes as indicated on each picture. Spots detected: (A) 682, (B) 227, (C) 662, (D) 640. Click here for file
