# Supplementary figures and images for: Optimisation of the two-dimensional gel electrophoresis protocol using the Taguchi approach
Source: Proteome Sci. 2004 Sep 9;2:6. doi: 10.1186/1477-5956-2-6 (PMC517948; doi:10.1186/1477-5956-2-6)

**(A)** 0.4%ASB14, 0.25% Ampholytes

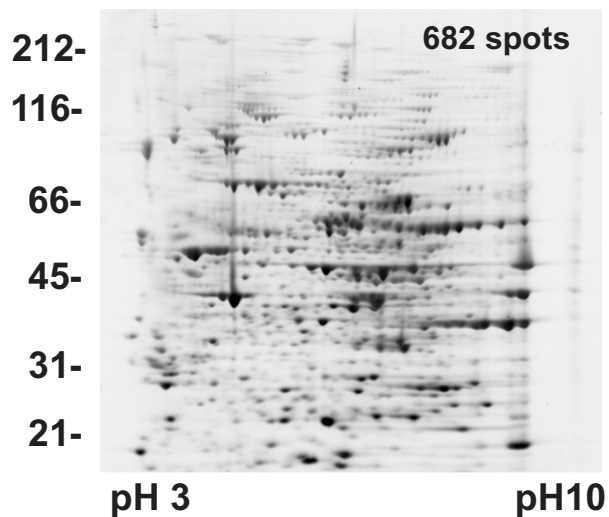

**(B)** 2.4%ASB14, 0.25% Ampholytes

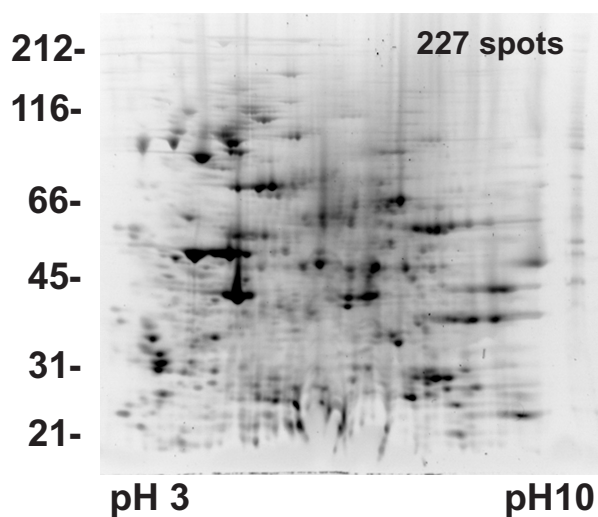

**(C)** 0.1%ASB14, 0.25% Ampholytes

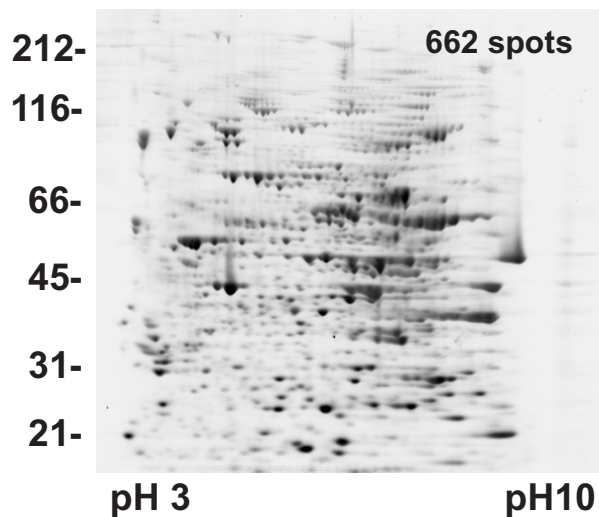

**(D)** 0.4%ASB14, 0.05% Ampholytes

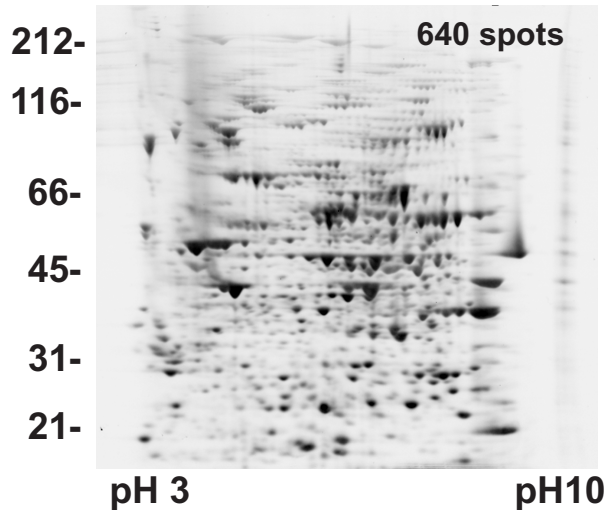

Supplement: Additional File 1 — 2DE response to variations of ASB14 and ampholytes concentrations. 50 μg aliquots of total Xenopus egg extract were dissolved in RBs containing 7 M urea, 2 M thiourea, 1.2% CHAPS, 43 DTT and variable amount of ASB14 and ampholytes as indicated on each picture. Spots detected: (A) 682, (B) 227, (C) 662, (D) 640. [file 1477-5956-2-6-S1.pdf]
